# Therapeutic Value of Combined Therapy with Deferasirox and Silymarin on Iron Overload in Children with Beta Thalassemia

**DOI:** 10.4084/MJHID.2013.065

**Published:** 2013-11-04

**Authors:** Adel A. Hagag, Mohamed S Elfrargy, Rana A. Gazar, Aml Ezzat Abd El-Lateef

**Affiliations:** 1 Department of Pediatrics, Faculty of Medicine, Tanta University, Egypt; 2 Department of Clinical Pathology, Faculty of Medicine, Tanta University, Egypt

## Abstract

**Background:**

Beta thalassemia is an inherited hemoglobin disorder resulting in a severe, chronic anemia requiring life-long blood transfusion that induces iron overload. Silymarin is a flavonoid complex isolated from Silybin marianum with a strong antioxidant activity, inducing an hepatoprotective action, and probably, a protective effect on iron overload. The aim of this work was to determine the silymarin value in improving iron chelation in thalassemic patients with iron overload treated with Deferasirox.

**Patients and Methods:**

This study was conducted on 40 children with beta thalassemia major under follow-up at Hematology Unit, Pediatric Department, Tanta University Hospital with serum ferritin level more than 1000 ng/ml and was divided into two groups. Group IA: Received oral Deferasirox (Exjade) and silymarin for 6 months. Group IB: Received oral Deferasirox (Exjade) and placebo for 6 months and 20 healthy children serving as a control group in the period between April 2011 and August 2012 and was performed after approval from research ethical committee center in Tanta University Hospital and obtaining an informed written parental consent from all participants in this study.

**Results:**

Serum ferritin levels were markedly decreased in group IA cases compared with group IB (P= 0.001).

**Conclusion:**

From this study we concluded that, silymarin in combination with Exjade can be safely used in the treatment of iron-loaded thalassemic patients as it showed good iron chelation with no sign of toxicity.

**Recommendations:**

We recommend extensive multicenter studies in a large number of patients with longer duration of follow-up and more advanced techniques of assessment of iron status in order to clarify the exact role of silymarin in reducing iron overload in children with beta thalassemia.

## Introduction

Thalassemias are a heterogeneous group of inherited anemias that collectively represents the most common monogenic disorders. The different forms of β-thalassemia are characterized by reduced or absent production of β-chains of hemoglobin. Patients with the most severe form of β-thalassemia major or Cooley’s present a profound anemia that, if not treated, leads to death in the first few years of life. The only available curative therapy is allogeneic bone marrow transplantation which is available for less than 30% of patients.^1^

The most common treatment for the most serious types of thalassemia is blood transfusion which is necessary, in order to provide the patient with healthy red blood cells containing normal hemoglobin. Repeated blood transfusion leads to iron overload.^2^ In iron overload excess iron accumulates in the body which is deposited in body organs as heart, liver and endocrine glands causing organ damage. Probably, iron saturates the liver firstly, and then accumulates in other organs. Excess iron accumulation is a leading cause of clinical deterioration and often death.^3^

The emergence of new iron chelators has a major impact on the treatment of thalassemia major. Moreover, the availability of more than one iron chelators opens up the possibility of reducing iron overload of specific organs while enhancing its overall excretion.^4^

Deferasirox*is* a triazole compound with two molecules of it are needed to bind one molecule of iron fully (tridentate chelator). It has high affinity to iron, with minimal binding to copper and zinc. It is supplied as orally dispersible tablets that are dissolved in water or juice and administered best on an empty stomach. Deferasirox- iron complex is excreted almost exclusively in the feces, with minimal urinary excretion.^5^ Deferasirox has been implemented as an alternative to the gold standard chelator, desferrioxamine.^6^ Deferasirox is administered, once-daily with a good safety and efficacy profile.^7^ It is marketed as Exjade and is mainly used is to reduce iron overload in patients who are receiving long-term blood transfusions as beta-thalassemia and other chronic anemias.^8^

Silymarinis is an herbal remedy used for the treatment of liver and gall bladder disorders. Silymarin is a flavonoid complex extracted from milk thistle (*Silybum marianum*). It has a strong antioxidant, hepatoprotective, and iron chelating antivities.^9^ There are some studies designed to investigate the therapeutic activity of orally administered silymarin in patients with thalassemia major under conventional iron chelation therapy.^10^

## Aim of the Work

The aim of this work was to compare the iron chelating efficacy of oral Deferasirox compared with combination therapy of oral Deferasirox and silymarin in children with beta thalassemia major with iron overload.

## Patient and Methods

This prospective, study was conducted on 40 children with beta thalassemia major under follow-up at Hematology Unit of Pediatric Department, Tanta University Hospital having serum ferritin level more than 1000 ng/ml and 20 healthy children serving as a control group in the period between April 2011 and August 2012 and was performed after approval from research ethical committee center in Tanta University Hospital and obtaining an informed written parental consent from all participants in this research.

### Study design

Thalassemic patients included in the study (group I) were divided into two subgroups, Group IA and Group IB, by simple random allocation; Group IA received combination of oral Deferasirox 20–40 mg/kg/day supplied in orally dispersible tablets that are dissolved in water or juice and administered best on an empty stomach^5^ with oral silymarin in the form of Legalon tablets 140 mg, one hour before each meal (3 times daily) for 6 months^11^ while group IB received oral Deferasirox 20–40 mg/kg/day and placebo. Group II: included 20 healthy children matched in age and sex serving as a control group.

#### Inclusion criteria will be

Children with β-thalassemia with serum ferritin > 1000 ng/ml who did not received any iron chelation therapy before the start of this study.

#### Exclusion criteria will be

Children with β-thalassemia with serum ferritin < 1000 ng/ml or who received any iron chelation therapy before the start of this study.

#### All the children in both groups were subjected to the following

1. Complete history taking with especial account on onset of thalassemia, chelation therapy, frequency of blood transfusion.
2. Through clinical examination with especial account on: pallor, jaundice, mongloid facies, splenomegaly, hepatomegaly and splenectomy.
3. Investigations including:
  - Complete blood count.
  - Hemoglobin electrophoresis.
  - Liver functions including bilirubin level, alanine transferase (ALT) and aspartate transferase (AST).
  - Renal function tests including blood urea and serum creatinine.
  - Assessment of serum iron status including serum ferritin, serum iron and iron binding capacity. Assessment of liver function tests, renal function tests and serum iron status were done two times in group I; one time before the start of chelation therapy and one time after chelation therapy but only one time in control group.

#### Specimen collection and handling

blood specimens were collected in a plain tube using sterile needles through gentle venipuncture after sterilization of site of puncture by alchol, and collected samples were allowed to clot for 4 minutes then centrifuged to separate clear non hemolysed serum.^12^

#### Determination of serum iron

The iron dissociated from transferrin-iron complex by a solution of guanidine acetate and reduced by ascorbic acid reacts with ferrozine to give a pink complex (according to procedure recommended by the serum iron from Biomaghreb company).^13^

#### Determination of serum total iron binding capacity (TIBC)

An excess of iron is added to the serum to saturate the transferrin. The unbound iron is precipitated with basic magnesium carbonate (according to procedure recommended by the serum total iron binding capacity from Biomaghreb company).^14^

#### Serum ferritin test

Serum level of ferritin by ELIZA [DRG® Ferritin ELISA (EIA-4292)].^15^

### Principle of the test

Anti-human-ferritin antibodies are bound to microwells. Ferritin, if present in diluted serum or plasma, bind to the respective antibody. Washing of the microwells removes unspecific serum and plasma components. Horseradish peroxidase (HRP) conjugated anti-human ferritin immunologically detects the bound specimen sample ferritin forming a conjugate/ferritin/antibody complex. Washing of the microwells removes unbound conjugate. An enzyme substrate in the presence of bound conjugate hydrolyzes to form a blue color. The addition of an acid stops the reaction forming a yellow end-product. The intensity of this yellow color is measured photometrically at 450 nm. The amount of color is directly proportional to the concentration of ferritin present in the original sample.^15^

## Statistics

Statistical presentation and analysis of the present study was conducted, using the mean, standard deviation and chi-square test by SPSS Version 16.^16^

## Results

Table 1 shows no significant difference between group IA and group IB regarding age, sex distribution, age of onset of beta thalassemia, frequency of blood transfusion and clinical manifestations.

Table 2 show no significant difference between group IA and group IB regarding CBC, significant lower RBCs indices in patients than control groups, significant higher T.L.C. and platelet count in patients group than control group and significant higher reticulocyte count in patient groups than control group. Significant (p < 0.05), highly significant (p < 0.01), t1 comparison between group IA and group IB, t2 comparison between group IA and control, t3 comparison between group IB and control.

Table 3 shows no statistically significant difference in serum creatinine, blood urea, AST and ALT between Group IA and Group IB before and after chelation therapy and no significant difference between patients groups and control group. No statistically significant difference in serum biliruben level between Group IA and Group IB before and after chelation therapy but there was significantly higher serum bilirubin level in patient groups than control group. t1 comparison between group IA and group IB, t2 comparison between group IA and control, t3 comparison between group IB and control and t4 comparison between the same group (as group IA before and after chelation therapy).

Table 4 shows that serum ferritin and serum iron levels is significantly higher in patients than control group and there were no statistically significant difference in serum ferritin and serum iron levels between group IA and group IB before start of chelation therapy while there was statistically significant difference between group IA and group IB after chelation therapy with lower level of serum ferritin and serum iron in group IA. Serum TIBC level is significantly lower in patients than control group and there was no statistically significant difference in serum TIBC level between group IA and group IB before start of chelation therapy while there was statistically significant differences between group IA and group IB after chelation therapy with higher level of serum TIBC group IA. t1 comparison between group IA and group IB, t2 comparison between group IA and control, t3 comparison between group IB and control and t4 comparison between the same group (as group IA before and after chelation therapy.

## Discussion

Thalassemias are one of the most common genetic disorders worldwide. It is the commonest cause of chronic hemolytic anemia in Middle East.^17^ In beta thalassemia major, impaired biosynthesis of beta-globin leads to accumulation of unpaired alpha-globin chain, shortened red cell life span and iron overload causing functional and physiological abnormalities in various organ systems.^18^

Silymarin is a flavonoid agent with antioxidant and free radical scavenging abilities. Silymarin also acts as an iron chelator by binding Fe (III). Despite the iron chelating activity of silymarin suggests its possible application in chelation therapy of iron overload; the biological effects of silymarin are different from other iron chelators, probably due to antioxidant activity of silymarin, which causes pro-oxidant effect via iron-catalyzed oxidation with subsequent generation of reactive oxygen species.^11^

This study was carried out on 40 children with β-thalassemia major under follow up in outpatient’s clinic of Pediatric Hematology Unit, Tanta University Hospital and 20 healthy children serving as a control group in the period between April 2011 and August 2012. Patients included in the study (group I) were subdivided into two subgroups (group IA and group IB) by simple random allocation, group IA received combination of exjade with silymarin while group IB used exjade and placebo.

This study shows that; the age of onset of β-thalassemia in studied cases ranged from 6 – 36 months with non-significant difference (P.value=0.886) in age of onset between group IA and group IB. These results are in agreement with Kattamis,^19^ who said that symptoms of β-thalassemia appear usually in the first year of life, at the time when the synthesis of γ-chains is not replaced by the synthesis of β-chains, with the mean age at presentation of 13 months and Cao, et al.,^20^ who found that beta thalassemia was recognized at age around 8 months in patients with transfusion-dependent beta thalassemia but at age of 2 years in non–transfusion dependent thalassemic children.

In our study; pallor and jaundice represent the most common presenting symptoms in studied cases of both groups while hepatomegaly and splenomegaly represent the most common presenting signs. This data are in agreement with Galanello and Origa,^21^ who noted that these clinical finding are due to chronic hemolysis, extramedullary erythropoiesis and iron overload.

In this study, serum ferritin level is significantly higher in patients than control groups. This is in agreement with Hershko,^22^ who demonstrated that; in thalassemia major, iron overload is the main outcome of multiple blood transfusions and inappropriately increased iron absorption associated with ineffective erythropoiesis.

In the current study, there were no significant differences in the initial serum ferritin levels between group IA and group IB but after regular chelation therapy, serum ferritin level is significantly lowered in group IA than group IB. This is in agreement with Gharagozloo et al.,^10^ who assessed the efficacy of silymarin and desferroxamine compared with desferroxamine alone in removing excess iron by serial measurement of serum ferritin levels in 48 patients and found that, at the beginning of the trial, all patients had comparable mean serum ferritin levels but after receiving silymarin plus desferrioxamine, the drop in serum ferritin was higher than in desferrioxamine treated patients, indicating that the combined therapy depleted iron stores more successfully than desferroxamine alone. Our study is not in agreement with Adibi, et al.,^23^ who found that silymarin did not cause significant changes in liver iron concentration and concluded that evaluating a longer course of treatment with this drug is thus suggested. Variation in results could be explained by different mode of evaluating iron reduction (serum ferritin in our study versus liver iron concentration in Adibi, et al., study and variation in severity of iron over load in both studies.

In this study, serum iron level is significantly higher in patient groups than control group while TIBC level is significantly lower in patient groups than control group. This is in agreement with Ghone et al,^24^ who stated that, the majority of the beta thalassemia major patients had significant increase of serum iron with significant decrease of total iron binding capacity and have severe anemia due to ineffective erythropoiesis which is a primary reason for iron overload. Thus, increased iron may increase the potential of oxidative injury to erythrocyte and cell organelles.

In the current study, there were no significant differences in the initial serum iron and TIBC levels in studied patients but after regular administration of chelation therapy, serum iron level is significantly lowered in group IA than group IB and TIBC is significantly increased in group IA more than group IB. This is in agreement with Wood, 25 who stated that serum iron is increased and TIBC is decreased in cases of beta thalassemia.

In this study, there were no significant differences in pre and post values of renal and liver function tests in patients groups before and after chelation therapy. This data is in agreement with Gharagozloo, et al.,^10^ who demonstrated that; thalassemic patients with severe iron overload can be safely treated with a combination of silymarin and desferrioxamine with no detectable abnormalities in complete blood count, liver or renal functions due to silymarin use.

## Conclusion

From this study we concluded that, silymarin in combination with exjade can be safely used in treatment of iron-loaded thalassemic patients. It showed good iron chelation with no signs of toxicity.

## Recommendations

Extensive multicenter studies in large number of patients with longer duration of follow up and more advanced methods of assessment of iron status is recommended to clarify the exact role of silymarin in reduction of iron over load in children with beta thalassemia.

## Figures and Tables

**Table 1:** Clinical data of studied thalassemic patients.

	Group IA (No=20)	Group IB (No=20)	Chi-Square	
			X^2^	P - value

**Age (years)**				
Range	2.5–6	2.5–6	0.144	0.886
Mean ± SD	5.17 ± 1.8	5.10 ± 1.16		

**Sex**				
Males	10	9	0.100	0.752
Females	10	11		

**Age of onset of thalassemia (months)**				
Range	6–36	6–36	0.100	0.921
Mean ± SD	10.85 ± 9.2	11.15 ± 9.76		

**Frequency of blood transfusion**				
Every 2 week	4 cases	5 cases		
Every 3 week	10 cases	5 cases	2.921	0.404
Every 4 week	5 cases	9 cases		
Every 6 week	1 case	1 case		

**Clinical manifestations**				
Pallor	16	18	0.253	0.224
Jaundice	15	13	1.635	0.414
Mongloid facies	3	2	1.00	0.523
Splenomegaly	19	16	0.855	0.669
Hepatomegaly	15	17	0.225	0.885
Splenoctomy	2	3	0.357	0.335

**Table 2:** Comparison of CBC between patients and control group.

		Patients		Controls (No=20)	T value p value	
		Group IA (No=20)	Group IB (No=20)			
**Hemoglobin (Hb) (gm/dl)**	Range	7–8	7–8.8	11–13	t1. 0.96 t2. 2.66 t3. 2.88	p1. 0.635 p2. 0.047 p3. 0.030
	Mean ± SD	7.3 ± 0.32	7.72 ± 0.60	11.9 ± 0.66		
**Mean Corpuscular Volume (MCV) (fl)**	Range	72.4–75.6	72.5–80.7	75–88	t1. 1.11 t2. 2.52 t3. 1.65	p1.0.114 p2.0.063 p3.0.010
	Mean ± SD	73.2 ± 1.01	76.2 ± 3.09	80.9 ± 3.79		
**Mean Corpuscular Hemoglobin (MCH) (pg)**	Range	23.5–25.9	23–26	26–34	t1. 0.52 t2. 1.04 t3. 1.88	p1. 0.253 p2. 0.063 p3. 0.044
	Mean ± SD	24.4 ± 0.66	25.1 ± 0.79	29.9 ± 2.4		
**Total leucocytic count (T.L.C) (thousands/mm^3^)**	Range	6.7–18.5	7–18	4–12	t1. 1.12 t2. 3.22 t3. 3.44	p1. 0.096 p2. 0.008 p3.0.006
	Mean ± SD	11.81 ± 3.91	10.74 ± 3.25	6.72 ± 2.51		
**Platelets (thousands/mm^3^)**	Range	160–688	170–688	150–430	t1. 0.75 t2. 4.52 t3. 3.22	p1. 0.114 p2. 0.030 p3. 0.018
	Mean ± SD	359.6 ± 32.45	357.65 ± 35.71	292 ±18.75		
**Reticulocytes %** **Retic. count (thousands/mm^3^)**	Range	4–7%	4–7%	0.5–1.6%	t1. 1.99 t2. 2.95 t3. 3.25	p1. 0.050 p2. 0.012 p3. 0.009
		170–384	220–350	275–880		
	Mean ± SD	311.2 ± 24.6	285.3 ± 32.7	517 ±15.5		

**Table 3:** Renal and hepatic function tests in studied patients and control group before and after chelation therapy.

		Group IA **(No=20)**	Group IB **(No=20)**	Control **(No=20)**	T value p value	
**Serum creatinine** (mg/dl)	Before	0.55±0.12	0.52±0.16	0.43±0.06	t1. 0.85 t2. 0.66 t3. 0.55	p1. 0.889 p2. 0.635 p3. 0.745
	After	0.58±0.15	0.60±0.15		t1. 0.44 t2. 0.63 t3. 0.25	p1. 0.256 p2. 0.478 p3. 0.625
	t. test	1.693	2.362			
	p. value	0.224	0.042			
**Blood urea** (mg/dl)	Before	28.55±6.98	30.40±6.78	25.63±5.63	t1. 1.63 t2. 1.22 t3. 1.74	p1. 0.635 p2. 0.114 p3. 0.227
	After	29.50±6.71	28.60±6.52		t1. 0.58 t2. 1.30 t3. 0.95	p1. 0.258 p2. 0.529 p3. 0.663
	t4 test	0.658	0.635			
	P4 value	0.442	0.158			
**ALT** (U/L)	Pre	17.50±3.17	18.80±4.53	15.63±2.66	t1. 0.52 t2. 0.33 t3. 0.44	p1. 0.635 p2. 0.263 p3. 0.419
	Post	18.25±3.17	18.90±3.68		t1. 0.75 t2. 0.62 t3. 0.44	p1. 0.324 p2. 0.752 p3. 0.620
	t. test	0.856	0.968			
	P. value	0.635	0.754			
**AST** (U/L)	Before	24.70±3.37	25.65±4.47	21.47±4.52	t1. 0.61 t2. 0.75 t3. 0.99	p1. 0.472 p2. 0.529 p3. 0.335
	After	25.05±4.67	24.71±3.79		t1. 0.44 t2. 0.80 t3. 0.93	p1. 0.475 p2. 0.841 p3. 0.669
	t4 test	1.366	0.998			
	p4 value	0.258	0.417			
**Total bilirubin** (mg/dl)	Before	2.99±1.24	3.11±0.52	0.50±0.15	t1. 1.36 t2. 2.15 t3. 2.88	p1. 0.425 p2. 0.069 p3. 0.056
	After	3.12±0.72	2.96±0.81		t1. 2.415 t2. 6.325 t3. 8.663	p1. 0.048 p2. 0.001 p3. 0.001
	t4 test	0.536	0.528			
	p4 value	0.442	0.358			

**Table 4:** Serum iron status in studied patients and control group before and after chelation therapy.

Parameters	Group IA (No=20)	Group IB (No=20)	Control (No=20)	t value	P value
**Ferritin (ng/ml)** Before chelation After chelation	3253.7±707.1	3049.2±527.7	203±56.7	t1. 1.03 t2. 12.52 t3. 15.63	p1. 0.307 p2. 0.001 p3. 0.001
	1067.2±297.9 t4 6.325 p4 0.001	1795.3±551.6 t4 3.669 p4 0.005		t1. 10.25 t2. 14.52 t3. 18.63	p1. 0.001 p2. 0.001 p3. 0.001
**Iron (ug/dl )** Before chelation After chelation	248.85±38.2	234.1±36.2	83.6±9.4	t1. 3.25 t2. 8.66 t3. 12.22	p1. 0.047 p2. 0.001 p3. 0.001
	137.4±31.1 t4 8.365 p4 0.001	178.15±40.14 t4 5.639 p4 0.005		t1. 6.33 t2. 18.66 t3. 15.22	p1. 0.001 p2. 0.001 p3. 0.001
**TIBC (ug/dl )** Before chelation After chelation	199.4±19.36	200.1±25.5	329±50.3	t1. 0.88 t2. 6.33 t3. 8.88	p1. 0.996 p2. 0.009 p3. 0.003
	332.1±36.5 t4 4.558 p4 0.007	259.2±24.1 t4 6.335 p4 0.001		t1. 2.33 t2. 3.15 t3. 8.99	p1. 0.039 p2. 0.003 p3. 0.008
